# Supplementary material for: “It’s not that I don’t trust vaccines, I just don’t think I need them”: Perspectives on COVID-19 vaccination
Source: PLoS One. 2024 Feb 15;19(2):e0293643. doi: 10.1371/journal.pone.0293643 (PMC10868828; doi:10.1371/journal.pone.0293643)
Supplement: S1 Appendix — (DOCX) [file pone.0293643.s001.docx]

**S1 Appendix. Focus Groups Interview Guides (French)**

**Guide d’entretien pour les groupes de discussion – parents d’enfants âgés entre 6 mois et 4 ans**

**Introduction (10 minutes)**

- Souhaiter la bienvenue aux participants
- Présenter le contexte de la rencontre et présenter les objectifs
- Présenter le déroulement de la rencontre: durée, enregistrement, confidentialité, observateurs, questions et commentaires, etc.
- Inviter les participants à se présenter: prénom, région, occupation, situation familiale (c*onjoint ou non, nombre d’enfants et leur âge*).
- Demander si certains participants ou leur(s) enfant(s) ont eu la COVID-19 depuis le début de la pandémie / depuis le début de l’année. Ne pas entrer dans les détails.
- Faire un premier tour de table pour recueillir brièvement les perceptions entourant la vaccination contre la COVID-19 : Spontanément, qu’est-ce qui vous vient en tête sur je dis les mots – **vaccination contre la COVID-19**? Ne vous inquiétez pas si quelqu’un a déjà mentionné ce que vous voulez nous dire lorsque nous serons rendus à vous; c’est normal. Cela signifie simplement que plusieurs d’entre vous ont le même genre d’idée sur cette question!

**(15 minutes) - Perceptions face à la COVID-19 et aux vaccins (efficacité, préoccupations quant à la sécurité, etc.)**

1. Quel est votre **niveau de confiance** envers les autorités de santé publique face aux décisions qui sont prises en ce qui concerne la vaccination contre la COVID-19?
2. Jusqu’à présent, que pensez-vous du **déroulement de la campagne de vaccination** contre la COVID-19?
3. Avez-vous des **préoccupations** concernant les vaccins contre la COVID-19? Si oui, quelles sont-elles?

**(25 minutes) - Intérêts et intentions face à la vaccination et barrières / facteurs facilitants anticipés**

La vaccination contre la COVID-19 est offerte aux enfants âgés entre 6 mois et 4 ans depuis la fin du mois de juillet.

1. Selon vous, quels sont les avantages ou les bénéfices de la vaccination des enfants âgés entre 6 mois et 4 ans?
2. Quels sont les risques ou les désavantages de la vaccination contre la COVID-19 des enfants âgés entre 6 mois et 4 ans?
3. Avez-vous l’intention de faire vacciner votre ou vos enfants? Pour quelle(s) raison(s)?
4. Pour ceux et celles qui hésitent/ne savent pas encore ou qui voudraient attendre avant de faire vacciner leur(s) enfant(s) :
   1. Qu’est-ce qui vous fait hésiter ?
   2. Qu’est-ce qui vous inciterait à accepter la vaccination ou qui vous ferait changer d’idée? Ou pouvez-vous pensez à une situation dans laquelle vous envisageriez de faire vacciner votre enfant? (par ex. nouvelles restrictions de voyage, passeport vaccinal à long terme, variant plus contagieux, etc.)
5. *Pour ceux et celles qui ont des enfants âgés de 5 à 11 ans ou de 12 à 17 ans (si cela se présente) : Avez-vous fait vacciner ou non ? Pour quelle(s) raison(s)?*
6. Concernant l’organisation des services de vaccination contre la COVID-19, quelles seraient les conditions idéales pour que la vaccination contre la COVID-19 pour votre famille? Qu’est-ce qui faciliterait les choses? Ou qui ferait en sorte que ce soit une expérience réussie? (explorer au besoin: des barrières possibles à l’accès/l’offre/la promotion de cette vaccination, par ex. le message véhiculé par les autorités, le nombre de lieux de vaccination, les horaires de vaccination, etc.)
7. Quels sont les enjeux/défis ou les difficultés que vous anticipez pour la vaccination? (explorer l’organisation des services – distance, lieux - et les préoccupations des enfants et des parents – peur de la douleur et des aiguilles, craintes des effets secondaires).

**(15 minutes) – Perceptions des vaccins de routine et impact de la pandémie et vaccination contre la COVID-19**

1. Comment la campagne de vaccination contre la COVID-19 a-t-elle influencé votre opinion concernant les autres vaccins recommandés au Québec?
2. Y a-t-il d’autres vaccins administrés aux enfants qui vous préoccupent? Si oui, lesquels? Quelles sont vos inquiétudes?
3. Est-ce que depuis le début de la pandémie, l’un de vos enfants n’a pas reçu un ou des vaccins recommandés? Si oui, lesquels et pour quelle(s) raison(s)?
   1. Avez-vous l’intention que votre enfant reçoive ce ou ces vaccins? Pourquoi?

**(20 minutes) - Sources d’information sur la COVID-19**

1. À quel point discutez-vous de la vaccination contre la COVID-19 avec des personnes de votre entourage (par ex. l’autre parent, la famille, amis, collègues, médecin de famille)? Et quel(s) sujet(s) abordez-vous le plus souvent?
2. Jusqu’à présent, que pensez-vous des informations disponibles sur la vaccination des enfants contre la COVID-19 ? (par ex. clarté de l’information, quantité d’information, etc.)
3. De quoi auriez-vous besoin pour prendre une décision (ou pour vous sentir plus confortable dans votre décision) concernant la vaccination contre la COVID-19 pour votre ou vos enfants?

Y-a-t-il autre chose que vous aimeriez partager avec nous au sujet de la vaccination contre la COVID-19?

**Conclusion (5 minutes)**

- Remercier les participants
- Prendre en note les questions des participants ainsi que les coordonnées afin de leur retourner une réponse
- Compensation

**Guide d’entretien pour les groupes de discussion – adultes au sujet de la dose de rappel**

**Introduction (10 minutes)**

- Souhaiter la bienvenue aux participants
- Présenter le contexte de la rencontre et présenter les objectifs
- Présenter le déroulement de la rencontre: durée, enregistrement, confidentialité, observateurs, questions et commentaires, etc.
- Inviter les participants à se présenter: prénom, région, occupation, situation familiale (c*onjoint ou non, nombre d’enfants et leur âge*).
- Demander si certains participants ou leur(s) enfant(s) ont eu la COVID-19 depuis le début de la pandémie / depuis le début de l’année. Ne pas entrer dans les détails.
- Faire un premier tour de table pour recueillir brièvement les perceptions entourant la vaccination contre la COVID-19 : Spontanément, qu’est-ce qui vous vient en tête sur je dis les mots – **vaccination contre la COVID-19**? Ne vous inquiétez pas si quelqu’un a déjà mentionné ce que vous voulez nous dire lorsque nous serons rendus à vous; c’est normal. Cela signifie simplement que plusieurs d’entre vous ont le même genre d’idée sur cette question!

**(15 minutes) - Perceptions face à la COVID-19 et aux vaccins (efficacité, préoccupations quant à la sécurité, etc.)**

1. Quel est votre **niveau de confiance** envers les autorités de santé publique face aux décisions qui sont prises en ce qui concerne la vaccination contre la COVID-19?
2. Jusqu’à présent, que pensez-vous du **déroulement de la campagne de vaccination** contre la COVID-19?
3. Avez-vous des **préoccupations** concernant les vaccins contre la COVID-19? Si oui, quelles sont-elles?

**(25 minutes) - Intérêts et intentions face à la vaccination et barrières / facteurs facilitants anticipés**

Dans les derniers mois, les autorités de santé ont incité la population à recevoir une dose de rappel du vaccin qui cible dorénavant le variant Omicron. Pour recevoir une dose de rappel, il est demandé que les personnes aient reçu leur dernière dose de vaccin depuis plus de 5 mois sans avoir contracté la maladie depuis.

1. Selon vous, quels sont les avantages ou les bénéfices de l’administration d’une dose de rappel du vaccin contre la COVID-19?
2. Quels sont les risques ou les désavantages de l’administration d’une dose de rappel du vaccin contre la COVID-19?
3. Combien de doses du vaccin avez-vous reçues jusqu’à présent? À quand remonte votre dernière dose reçue?
4. Pour quelle(s) raison(s) avez-vous reçu une dose de rappel? Pour quelle(s) raisons n’avez-vous pas reçu de dose de rappel? Avez-vous l’intention de recevoir une dose de rappel? Pour quelle(s) raison(s)?
5. Pour ceux et celles qui hésitent/ne savent pas encore ou qui voudraient attendre avant de recevoir une dose additionnelle du vaccin :
6. Qu’est-ce qui vous fait hésiter ?
7. Qu’est-ce qui vous inciterait à accepter la vaccination ou qui vous ferait changer d’idée? Ou pouvez-vous penser à une situation dans laquelle vous envisageriez de recevoir une nouvelle dose du vaccin? (par ex. passeport vaccinal à long terme, variant plus contagieux, etc.)
8. *Pour ceux et celles qui ont des enfants âgés de 5 à 11 ans ou de 12 à 17 ans (si cela se présente) : Est-ce que vos enfants ont été vaccinés contre la COVID-19 jusqu’à présent et ont-ils eu une dose de rappel ? Pour quelle(s) raison(s)?*
9. Quels sont les enjeux/défis ou les difficultés que vous anticipez pour l’administration de doses de rappel? (explorer l’organisation des services – distance, lieux et les craintes liées aux effets secondaires.

**(15 minutes) – Perceptions des vaccins de routine et impact de la pandémie et vaccination contre la COVID-19**

1. Comment la campagne de vaccination contre la COVID-19 a-t-elle influencé votre opinion concernant les autres vaccins recommandés au Québec?
2. Y a-t-il d’autres vaccins qui vous préoccupent (par ex. grippe saisonnière)? Si oui, lesquels? Quelles sont vos inquiétudes?

**(20 minutes) - Sources d’information sur la COVID-19**

1. À quel point discutez-vous de la vaccination contre la COVID-19 avec des personnes de votre entourage (par ex. l’autre parent, la famille, amis, collègues, médecin de famille)? Et quel(s) sujet(s) abordez-vous le plus souvent?
2. Jusqu’à présent, que pensez-vous des informations disponibles sur la vaccination contre la COVID-19 et plus particulièrement au sujet de la dose de rappel? (par ex. clarté de l’information, quantité d’information, etc.)
3. De quoi auriez-vous besoin pour prendre une décision (ou pour vous sentir plus confortable dans votre décision) concernant l’administration d’une dose de rappel?

Y-a-t-il autre chose que vous aimeriez partager avec nous au sujet de la vaccination contre la COVID-19?

**Conclusion (5 minutes)**

- Remercier les participants
- Prendre en note les questions des participants ainsi que les coordonnées afin de leur retourner une réponse
- Compensation
